# Enhanced *in-vitro* anti-*Candida* efficacy of *Euphorbia milii* Des Moul mediated copper nanoparticles against clinically isolated *Candida albicans*

**DOI:** 10.22034/cmm.2024.345176.1493

**Published:** 2023-12

**Authors:** Rosy Bala, Narinder Kaur, Nitin Gupta, Shahbaz Aman, Shalini Shriwastav

**Affiliations:** 1 Department of Microbiology, Maharishi Markandeshwar Institute of Medical Sciences and Research, Maharishi Markandeshwar (Deemed to be University), Mullana, Ambala- Haryana, India; 2 Department of General Medicine, Maharishi Markandeshwar Institute of Medical Sciences and Research, Maharishi Markandeshwar (Deemed to be University), Mullana, Ambala-Haryana, India

**Keywords:** Anti-*Candida* nanoparticles, Antifungal resistance, Candidemia, Green synthesized copper nanoparticles

## Abstract

**Background and Purpose:**

Emergence of fungi as a pathogenic threat presents a significant challenge to public health, notably in intensive care units (ICUs) and among immunocompromised patients. Various factors, including sepsis-induced barrier disruptions, immune system dysfunction, and extremes of age, contribute to increased susceptibility to fungal infections. Hospital practices, such as prolonged surgeries, broad-spectrum antibiotic use, and invasive procedures, further exacerbate the risk. Fungal bloodstream infections, particularly those caused by *Candida albicans*, rank among the most common hospital-acquired infections, leading to substantial morbidity and mortality. The global rise in invasive candidiasis, particularly due to non-*albicans Candida* species, presents challenges in the diagnosis and treatment due to nonspecific symptoms and emerging antifungal resistance. Nanotechnology interventions particularly by utilizing green synthesized copper nanoparticles could possibly provide a novel solution to combat microbial colonization, biofilm formation, and drug resistance. This study aimed to assess the prevalence of candidemia, identify the distribution of causative *Candida* species, and understand their susceptibility patterns to commonly used antifungal agents for effective management in ICU settings. Additionally, the study sought to explore the *in vitro* anti-*Candida* activity of green copper nanoparticles synthesized using *Euphorbia milii* des moul extract.

**Materials and Methods:**

This study was conducted at Microbiology Laboratory of Maharishi Markandeshwar Institute of Medical Sciences and Research from January to December 2022, focused on ICU patients suspected of bloodstream infections. Blood samples were collected aseptically and processed using BD BACTECTM culture vials. Identification of organisms was performed via the Vitek-2 system by confirming candidemia with positivity in both blood samples. After that antifungal susceptibility testing was also performed against Clinical and Laboratory Standards Institute recommended antifungal drug using Vitek 2 system. G-CuNPs were synthesized using *E. milii* Des moul extract and possessed for physiochemical characterization. The anti-*Candida* activity of G-CuNPs was evaluated through the MTT assay and time kill assay. After that generation of intracellular reactive oxygen species and DNA degradation were evaluated to understand its mechanism.

**Results:**

This study identified a candidemia rate of 7.3% (58/789). Age and gender analysis revealed higher *Candida* colonization rates in individuals above 60 years old and females. Antifungal sensitivity profiling indicated notable resistance to fluconazole (27.59%) and voriconazole (25.86%). Synthesizing G-CuNPs using *E. milii* des moul extract represents a novel approach exhibiting significant fungicidal potency against clinically isolated *C. albicans*, supporting potential therapeutic applications.

**Conclusion:**

the findings concluded that synthesized G-CuNPs have tremendous potential to battle against medical device-borne infections by surface coating.

## Introduction

The emergence of fungi as a pathogenic threat posed a significant public health challenge, particularly in intensive care units (ICUs) and among immunocompromised patients, leading to heightened morbidity and mortality [ 1 ]. Many factors, such as sepsis-induced barrier disruptions, neutrophil dysfunction, impaired cell-mediated immunity, metabolic dysfunction, and extremes of age, compromise the defenses of the body, elevating susceptibility to fungal infections. Contributing factors include prolonged surgeries, broad-spectrum antibiotic use, cytotoxic chemotherapies, immunosuppressant utilization in transplantation, intravenous nutrition, multiple-lumen catheter use, renal replacement therapy, and mechanical ventilation [ 2 ]. Although intensive care units (ICUs) make up a small portion (around 5-10%) of total hospital beds, about 20% of ICU-admitted patients acquire nosocomial infection during their hospital stay [ 3 ]. Fungal bloodstream infections, particularly those due to *Candida albicans*, are recognized as major culprits in hospital-acquired fungal infections, ranking seventh among nosocomial pathogens according to the Centers for Disease Control [ 4 ]. The global surge in invasive candidiasis since the early 1990s has witnessed a shift towards non-albicans *Candida* (NAC) isolates, associated with treatment failures and higher mortality rates, including *C. glabrata*, *C. parapsilosis*, *C. tropicalis*, and *C. krusei*. Prevalence of invasive candidiasis in ICU patients is exacerbated by indwelling devices, facilitating the colonization of pathogenic microbes, particularly *Candida* species [ 5 ]. Early diagnosis of invasive candidiasis is challenging due to nonspecific clinical presentations overlapping with bacterial infections. Moreover, the lack of well-defined criteria for initiation of empirical antifungal therapy adds complexity [ 6 ]. Despite challenges, antifungal agents, primarily fluconazole, are frequently employed in ICU for patients unresponsive to antibacterial therapy, and this may result in fluconazole resistance [ 7 ]. Rising antifungal resistance underscores the importance of judicious antifungal prophylaxis. To address microbial colonization, biofilm development, and drug resistance, identifying novel, cost-effective, and environmentally friendly antimicrobial agents is imperative [ 8 ]. Nanotechnology interventions, particularly utilizing copper nanoparticles (Cu-NPs), show promise in nanoscience and nanomedicine disciplines, demonstrating antimicrobial, anticancer, and antioxidant properties. Green synthesis methods using plant extracts as reducing or capping agents offer advantages, such as stability, easy synthesis, enhanced production rates, cost-effectiveness, and the generation of non-toxic by-products [ 9 ]. This study aimed to assess the prevalence of candidemia, distribution of causative *Candida* species, and understand their susceptibility patterns to commonly used antifungal agents for effective management. Additionally, *in vitro* evaluations of the anticandidal activity of green copper nanoparticles synthesized using *Euphorbia milii* des moul extract were explored.

## Materials and Methods

### Study duration and setting

The study was conducted at the Microbiology Laboratory of Maharishi Markandeshwar Institute of Medical Sciences and Research, Mullana, India, from January 2022 to December 2022. Blood samples were collected from the patients admitted to ICUs and suspected of bloodstream infections after obtaining clearance from the Ethical Committee (IEC-119/XP) of Maharishi Markandeshwar Institute of Medical Science and Research, MMDU, Mullana, India.

### Blood sample collection and processing

Two separate blood samples were aseptically collected in BD BACTEC^TM^ (Dickinson and Company, USA) culture vials from each suspected patient. Samples showing positivity in BACTEC were sub-cultured on blood agar, MacConkey agar, and Sabourauds Dextrose Agar (Purchased from Himedia, India) followed by incubation at 37 °C for 24-48 h. Identification of the cultured organisms was performed using the Vitek-2 system. Candidemia was confirmed only when both blood samples from a single patient showed positivity for the same organism [ 10 ].

### Germ tube test

The germ tube test was conducted by inoculating 0.5 ml of pooled human serum with a *Candida* spp. colony. After incubation at 37 °C for 2-4 h, a drop of serum was transferred onto a glass slide and observed microscopically, and the presence of five or more germ tubes was considered significant for *C. albicans* [ 11 ].

### Antifungal susceptibility testing

Antifungal susceptibility testing was performed using the Vitek 2 automated system. The Vitek 2 cards containing serial twofold dilutions of amphotericin B, fluconazole, flucytosine, and voriconazole were provided by the manufacturer. Fungal suspension of 2 McFarland standard was prepared in a sterile polystyrene test tube, and the inoculum suspensions were diluted appropriately by the instrument, after which the cards were ﬁlled, incubated, and read automatically. The incubation time varied from 10 to 24 h based on the rate of growth in the drug-free control well [ 12 ].

### Synthesis and characterization of Euphorbia milii Des moul mediated copper nanoparticles

The current investigation made use of G-CuNPs that had already been produced and described. To summarize, a plant taxonomist from the Department of Botany at Sri Venkateswara University Tirupati in India, identified the plant as *Euphorbia Milli* Des moul after it was obtained from a medical garden at Maharishi Markandeshwar (deemed to be university) in Mullana, India (IAAT: 337). In the environment-friendly production of copper nanoparticles, the plant extract was synthesized using the soxhlet process and then utilized as a capping agent. Stirring distilled water containing dissolved CuSO4 and *E. milii* des moul extract was required until a color change was observed. To test for bio-reduction, the green-produced CuNPs were centrifuged, rinsed, and dried. Electron diffraction, particle/zeta analysis, and energy-dispersive X-ray analysis were used to assess the crystalline structure of the nanoparticles. The electron microscope was also used to study the intricate structure of the nanoparticles [ 13 ].

### Anti-Candida activity of G-CuNPs by using MTT

To assess the anti-*Candida* activity of G-CuNPs, a well-established colorimetric assay, the 3-(4,5-dimethylthiazol-2-yl)-2,5-diphenyltetrazolium bromide (MTT) assay (manufactured by Himedia, India), was employed. *Candida albicans* were cultured in Sabouraud Dextrose Broth until reaching the mid-log phase, and then the cells were treated with varying concentrations of G-CuNPs and incubated for 24 h at 37 °C. After incubation, the treated and untreated *C. albicans* cells were incubated with MTT (20mg/ml) for 4 h at 37 °C. The formed purple formazan crystals were solubilized in dimethyl sulfoxide, and the absorbance was measured at a wavelength of 570 nm using a microplate reader. The MTT assay measures cell viability based on the metabolic reduction of MTT by viable cells. A decrease in absorbance indicates reduced cell viability, thereby reflecting the antifungal activity of G-CuNPs. All experiments were performed in triplicate and statistical analyses were carried out to determine the significance of the observed effects [ 14 ].

### Time kill assay

The colony-forming unit count method was used to assess the anti-*Candida* activity of G-CuNPs (CFU). To summarize, 5 mg/ml of G-CuNPs were added to a 2 McFarland standard fungal suspension, and then the mixture was incubated at 37 °C. Likewise, the 2 McFarland standard fungal suspension that was treated with Fluconazole (2 µg/ml) was considered the positive control, whereas the 2 McFarland standard fungal suspension that was left untreated was considered the negative control. Following that, 100 µL of fungal suspension was collected and cultivated on Mueller–Hinton agar at 0-h, 3-h, and 6-h intervals. The mix was subsequently incubated at 37 °C for a whole day. Finally, CFU counts of fungal strains treated with G-CuNPs were compared to those of untreated (negative control) and fluconazole-treated fungal strains (positive control) [ 13 ].

### Determination of intracellular reactive oxygen species in Candida albicans

Reactive oxygen species (ROS) levels of yeast cells treated with G-CuNPs were measured using an intracellular ROS indicator known as Dichlorodihydrofluorescein diacetate (H2DCF-DA) (manufactured by Sigma Aldrich, USA). *Candida* strains with a light optical density (OD_600_) of 0.1 were exposed to a 5 mg/ml concentration of G-CuNPs and E. milii des moul extract. A positive control with 100 μM H2O2 was maintained for 1.5 h, and a negative control with 0.1 OD of yeast cells was accounted for. The cells were then rinsed with phosphate-buffered saline and left to incubate with 20 μM H2DCF-DA at 37 °C for 30 min. Finally, microplate readers (Shimadzu UV 2600 UV-Vis, Japan) were used to quantify fluorescence at 490 nm (excitation) and 520 nm (emission) after washing the treated cells [ 15 ].

### DNA degradation assay

Various components of the fungal cell, including genomic DNA, or protein, or fatty acid could be hampered by copper nanoparticles. Therefore, the authors further wanted to test the ability of G-Cu NPs and *E. milii* des moul extract to degrade the genomic DNA. For this, 0.1 OD of cells were treated with 5 mg/ml concentration of G-CuNPs and *E. milii* des moul extract for 24 h at 37 °C. In the case of negative control, untreated *C. albicans* were taken. Afterward, DNA was isolated using standard procedures and evaluated by agarose gel electrophoresis [ 16 ].

## Results

### Prevalence of pathogenic microorganisms

During the study period, 22.98% (789 out of 3432) of blood cultures revealed the growth of pathogenic microorganisms. Among these isolates, 58 isolates were identified as *Candida* spp., while the remaining isolates had a bacterial origin (Table 1).

**Table 1:** Distribution of isolates obtained from intensive care unit patients diagnosed with blood stream infection

Gram positive cocci		261
Gram negative bacilli		470
Yeast	*Candida parapsilosis*	1
	*Candida glabrata*	2
	*Candida famata*	3
	*Candida ciferrii*	6
	*Candida krusei*	7
	*Candida tropicalis*	9
	*Candida albicans*	30
**Total isolates**		**789**

### Distribution of candidemia cases in terms of age and gender

All Candida isolates were exclusively obtained from ICU patients. The age distribution indicated that the majority of them (36 out of 58) were above 60 years old, while two cases were newborns and the remaining 20 cases fell within the 30-60 years age group. Gender-wise prevalence revealed that 31 *Candida*-positive cases were noted in female patients while 27 were identified in males.

### Antifungal susceptibility testing

The antifungal susceptibility profile of the Candida isolates is represented in Table 2. Each row corresponds to a specific *Candida* species, including *C. albicans*, *C. ciferrii*, *C. famata*, *C. glabrata*, *C. Krusei*, *C. parapsilosis*, and *C. tropicalis*. For each species, Table 2 displays the number of isolates tested that were either sensitive or resistant to different antifungal agents recommended as per Clinical and Laboratory Standards Institute guidelines [ 17 ]. 

**Table 2:** Antifungal sensitivity profile of *Candida* species

*Candida* species (no.)	Amphotericin B		Caspofungin		Flucytosine		Fluconazole		Micafungin		Voriconazole	
	S(no.)	R(no.)	S(no.)	R(no.)	S(no.)	R(no.)	S(no.)	R(no.)	S(no.)	R(no.)	S(no.)	R(no.)
*C. albicans* (30)	25	5	26	4	27	3	27	3	26	4	21	9
*C. ciferrii* (6)	2	4	5	1	5	1	2	4	4	2	5	1
*C. famata* (3)	3	0	3	0	3	0	3	0	3	0	3	0
*C. glabrata* (2)	2	0	2	0	2	0	2	0	2	0	2	0
*C. krusei* (7)	5	2	7	0	5	2	0	7	6	1	5	2
*C. parapsilosis* (1)	1	0	1	0	1	0	1	0	1	0	1	0
*C. tropicalis* (9)	9	0	9	0	9	0	7	2	9	0	6	3
Overall sensitivity and Resistance (%)	81.03%	18.97%	91.38%	8.62%	91.38%	8.62%	72.41%	27.59%	87.94%	12.06%	74.14%	25.86%

S: Sensitive, R: Resistant

It was found that out of 30 *C. albicans* strains, 21 isolates were sensitive to voriconazole, 25 were sensitive to amphotericin B, 26 isolates were sensitive to caspofungin and micafungin and 27 isolates were sensitive to flucytosine and fluconazole. In the case of *C. ciferrii*, out of six isolates, five isolates were sensitive to caspofungin and flucytosine, and four isolates were sensitive to micafungin, whereas two isolates were sensitive to Amphotericin B and fluconazole. In the case of *C. famata*, *C. glabrata*, and *C. parapsilosis*, all isolates were sensitive against all tested antifungal agents. In the case of *C. Krusei*, all of the seven isolates were sensitive to caspofungin, six isolates were sensitive to micafungin, five isolates were sensitive to amphotericin B, flucytosine, and voriconazole, whereas no isolates were sensitive to fluconazole. In the case of *C. tropicalis*, all isolates were sensitive to amphotericin B, caspofungin, flucytosine, and micafungin, seven isolates were sensitive to fluconazole, and six isolates were sensitive to voriconazole. Overall, caspofungin and flucytosine were found to be the most effective antifungals with a sensitivity rate of 91.38%, whereas fluconazole was found to be the least effective with a resistance rate of 27.59%. This information is crucial for understanding the efficacy of antifungal treatment options and guiding therapeutic interventions.

### Characterization of G-CuNPs

**Figure 1:**
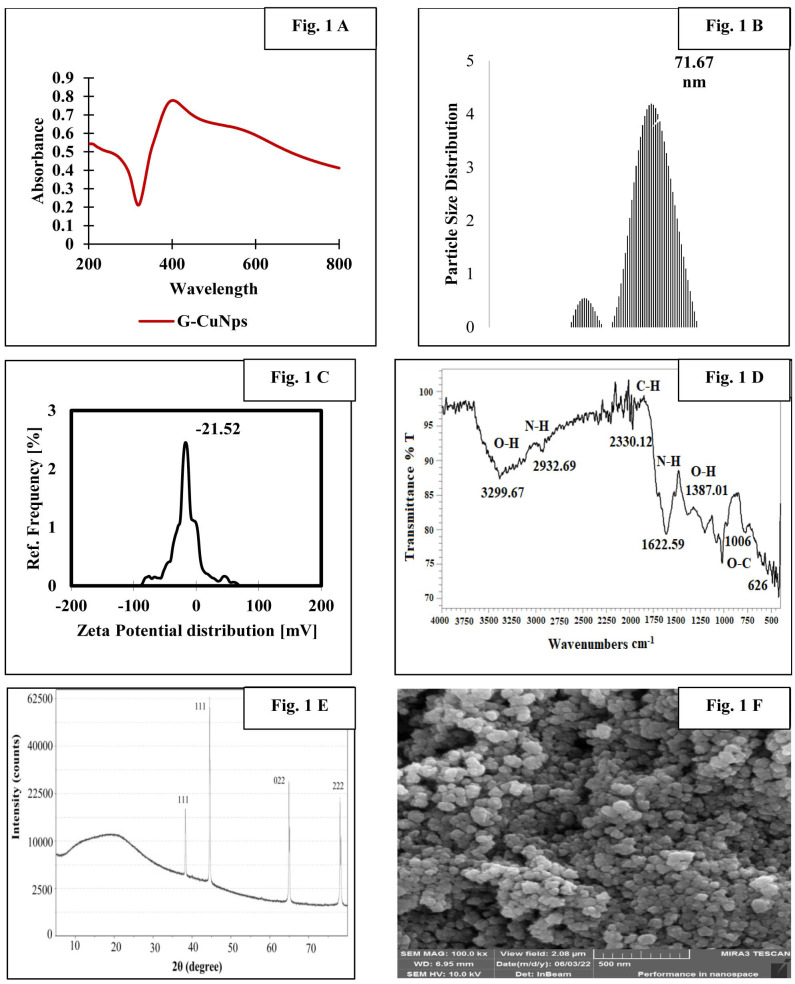
*Physio-Chemical characterization of G-CuNPs* *(A)* UV-Vis spectra analysis indicating synthesis of G-CuNPs; *(B)* The dynamic light scattering particle size analysis confirmed that the monodisperse G-CuNPs had an average radius of about 71.67 nm; *(C)* Zeta potential of G-CuNPs was found to be -21.52 mV; *(D)* Fourier transform infrared spectroscopy analysis of G-CuNPs; *(E)* X-ray diffraction pattern confirmed the Crystalline structure of G-CuNPs; *(F)* Transmission electron microscopic image showing the size distribution of spherical G-CuNPs ranging from 17 to 62 nm with an average size of 43.68 nm observed at 10 nm and 20 nm magnification.

By monitoring changes in surface plasmon resonance and confirming this with UV-visible spectroscopy, the study showed that Cu ions were being reduced to CuNPs (Figure 1 A). The surface charge of G-CuNPs was -21.52 mV, and their average particle size distribution was 71.67 nm (Figure 1 B) (Figure 1 C). O-H, N+-H, C-H, N-H, O-H, O-C, and aromatic H-banding were indicated by bands at 3299.67, 2932.69, 2330.12, 1622.59, 1387.01, 1006.41, and 626.60 in the Fourier transform infrared spectroscopy of G-CuNPs, respectively (Figure 1 D). Presence of three distinct peaks in the 2θrange of 38.36°, 44.47°, 64.80°, and 77.62° in the X-ray diffraction pattern of G-CuNPs—the 111, 022, and 222 reflection planes, respectively, confirms the crystallinity of the NPs (Figure 1 E). The G-CuNPs analyzed by transmission electron microscopy were uniformly sized and spherical, with an average size of 47 nm (Figure 1 F).

### Anti-Candida activity of G-CuNPs by using MTT

**Figure 2:**
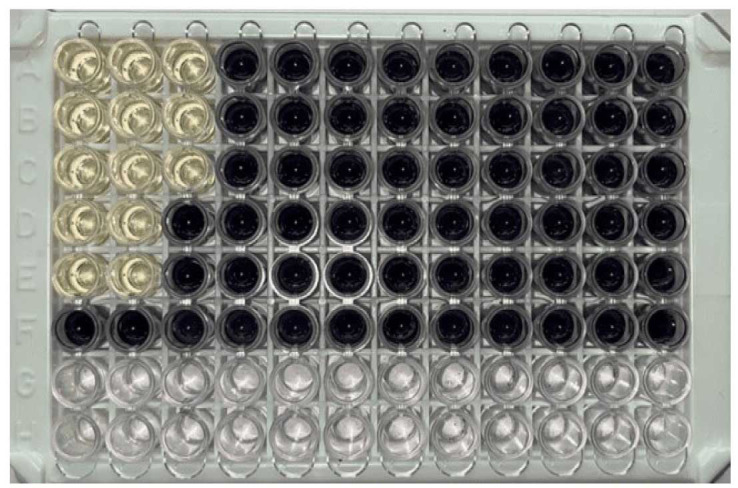
Minimum inhibitory concentration determination by micro broth dilution method using MTT dye

To understand the complex antifungal dynamics of G-CuNPs against *C. albicans*, the MTT assay was pivotal. The results showed that the antifungal action of G-CuNPs was concentration-dependent and gave important information about the dosage at which these nanoparticles were completely effective against *Candida*. The anti-*Candida* impact of G-CuNPs was particularly strong and all-encompassing at a concentration of 5 mg/ml. A considerable decrease in absorbance, according to the MTT assay, which measured the reduction of MTT by metabolically active cells, indicated reduced *C. albicans* viability. Moreover, no antifungal effect was observed at concentrations lower than 5 mg/mL. This was a very important finding since it showed that the antifungal effects of G-CuNPs were concentration-dependent. A complex interaction between G-CuNPs and *C. albicans* was supported by their inertness at lower concentrations; hence, an ideal dose was required to achieve significant antifungal effects. The results were further supported by the comparison with fluconazole, the standard antifungal medication. Treatment with fluconazole at a dosage of 2 µg/ml showed a significant result by entirely preventing the growth of *Candida*. This finding highlighted the similar effectiveness of G-CuNPs and the recognized antifungal drug and also acted as a positive control for the MTT experiment. At a dose of 5 mg/ml, G-CuNPs have antifungal properties similar to 2 μg/ml fluconazole, based on the equivalence in their inhibitory effect on *Candida* growth (Figure 2 and Table 3). Figure 2 displays the results of the MTT dye assay to determine the lowest inhibitory concentration of G-CuNPs against *C. albicans*. Table 3 shows the configuration of the microtiter wells with the concentration of fluconazole and G-CuNPs. When *C. albicans* development is seen in red, it means that the growth is uninhibited, whereas green indicates that it is completely inhibited.

**Table 3:** Minimum inhibitory concentration determination by microbroth dilution method using MTT dye

		1	2	3	4	5	6	7	8	9	10	11	12
G-CuNPs concentration (mg/ml)													
A	Candida albicans	20	10	5	2.5	1.25	0.625	0.312	0.156	0.078	0.039	0.019	0.009
B		20	10	5	2.5	1.25	0.625	0.312	0.156	0.078	0.039	0.019	0.009
C		20	10	5	2.5	1.25	0.625	0.312	0.156	0.078	0.039	0.019	0.009
**Antifungal concentration (µg/ml)**													
D	Fluconazole	4	2	1	0.5	0.125	0.062	0.031	0.015	0.007	0.004	0.001	0.0001
E		4	2	1	0.5	0.125	0.062	0.031	0.015	0.007	0.004	0.001	0.0001
F	PC	F/S	F/S	F/S	F/S	F/S	F/S	F/S	F/S	F/S	F/S	F/S	F/S
G	-----------	-----	-----	-----	-----	-----	-----	-----	-----	-----	-----	-----	-----
H	-----------	-----	-----	-----	-----	-----	-----	-----	-----	-----	-----	-----	-----

### Time kill assay

**Figure 3:**
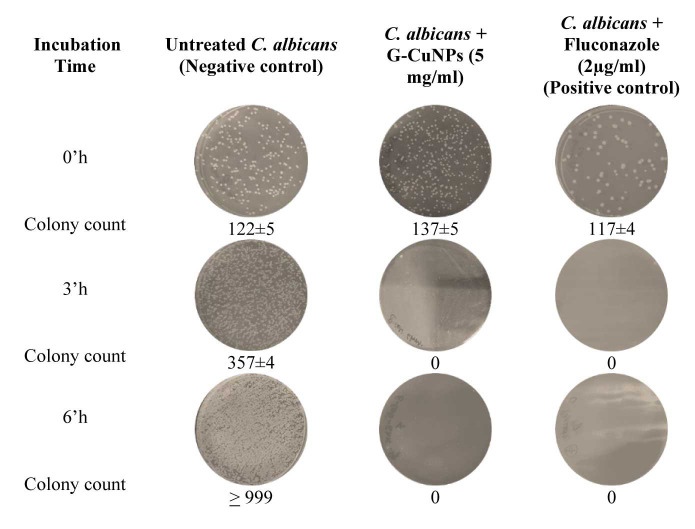
Anti-*Candida* efficacy of G-CuNPs tested by colony-forming units’ method against clinically isolated *Candida albicans* compared to positive and negative control.

The present study used the CFU count method to investigate the anti-*Candida* potency of G-CuNPs. After 6 h of incubation against the tested organism, G-CuNPs at a dosage of 5 mg/ml showed fungicidal action. Likewise, in the positive control group, *C. albicans* was completely eliminated within 3 h of administration of 2 µg/ml of fluconazole, whereas in the negative control group, *C. albicans* showed a progressive increase in CFU without treatment (Figure 3). The data shown here are the means values from three independent runs of the experiment. According to the Mann-Whitney test (*P*<0.05), the variations were statistically significant.

### Reactive oxygen species accumulation and DNA degradation

Using H2DCF-DA, the ability of G-CuNPs to generate ROS in *C. albicans* was assessed. *Candida albicans* supplemented with G-CuNPs showed an increase in fluorescence level after treatment with H2DCF-DA, suggesting the formation of ROS. In comparison to *C. albicans* treated with H_2_O_2_, all of the strains supplemented with G-CuNPs produced ROS at a similar rate (positive control) (Figure 4 A). The genomic stability of G-CuNPs treated with *C. albicans* was also assessed using gel electrophoresis. Genetic deterioration was observed in G-CuNPs but not in the extract, which is quite intriguing (Figure 4 B).

**Figure 4:**
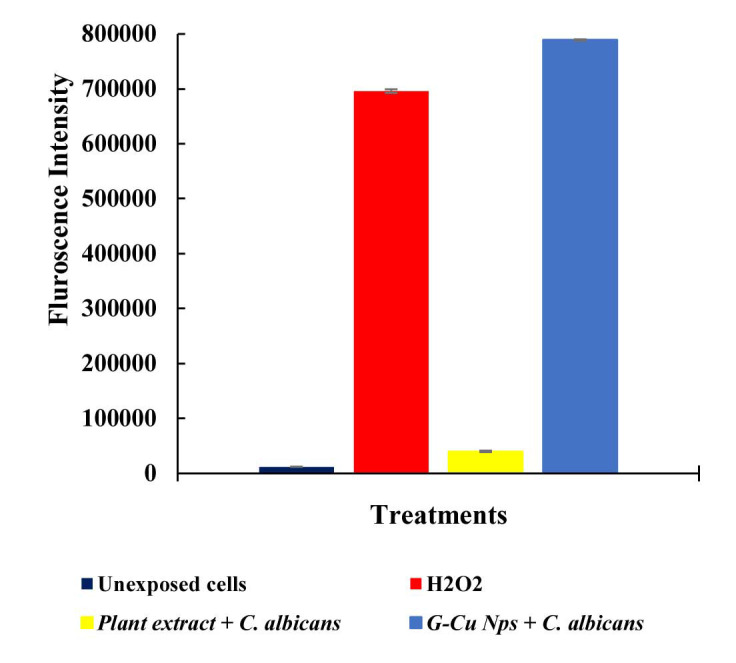
**A** ROS accumulation assay

**Figure 4:**
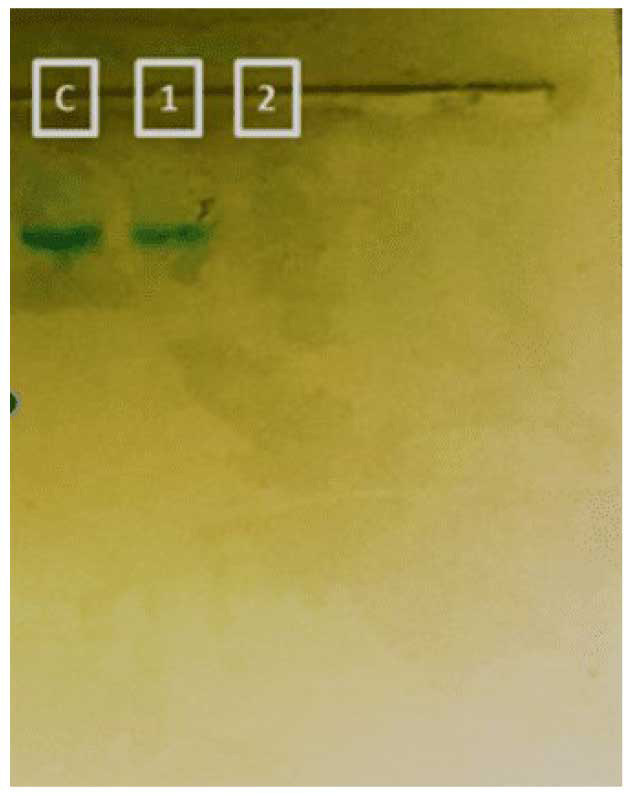
B DNA degradation assay (**C:** Candida albicans without any treatment; **1:** *C. albicans* treated with *E. milii* des moul extract; **2:** *C. albicans* treated with G-CuNPs)

## Discussion

The present study delved into the prevalence of candidemia among ICU patients, offering a comprehensive examination of *Candida* species distribution, antifungal susceptibility patterns, and the *in vitro* anti-*Candida* activity of G-CuNPs. Candidemia, a global concern with substantial morbidity and mortality rates, particularly impacts immunocompromised individuals. The observed shift from *C. albicans* to non-albicans species, notably *C. tropicalis* and *C. parapsilosis*, is in line with emerging trends reported in India [ 18 ]. The identified candidemia rate of 7.3% (58/789) corresponded closely with that of another study which was 7.76% among 1,440 cases [ 19 ] and diverged notably from the findings of another research which reported a higher prevalence of about 14.8% among 225 cases [ 20 ]. Another study conducted in northern India showed a prevalence rate of 0.34% for candidemia, that is 116 cases among 33,865 patients [ 21 ]. 

Increased prevalence of candidemia could be attributed to factors, such as variations in patient populations, geographical locations, or hospital-specific practices. Other contributing factors include compromised immune systems, extended hospital stays, and infection control measures. A study performed in northern India revealed that 3.41% of the blood cultures were positive for Candida species, while 35.21% of the isolates were C. tropicalis followed by other non-Candida albicans which accounted for about 50.71% of the isolates and C. albicans that caused 14.08% of the isolates [ 22 ]. Similarly, another study from north India reported a 2.8% (95/3,443) rate of candidemia prevalence, whereas C. tropicalis and C. parapsilosis accounted for 38% and 18% of the isolates, respectively [ 23 ]. Analysis of candidemia patients in terms of age and gender revealed higher *Candida* colonization rates in individuals above 60 years old, which corresponded with the established literature, emphasizing age as a factor influencing immune defense [ 24 ]. Antifungal sensitivity profiling, indicating higher resistance to fluconazole (27.59%) and voriconazole (25.86%), is consistent with the literature, supporting the notion that resistance to these antifungals is prevalent. Increased resistance of *Candida* species to fluconazole and voriconazole is logically linked to the potential overexpression of multidrug-resistant genes, impacting susceptibility [ 25 ]. The green synthesis method is used for nanoparticle preparation in the recent advancement of the nanomedicine field. In green synthesis, various biological sources could be utilized as reducing and capping agents for the synthesis of nanoparticles [ 26 ]. The synthesis of G-CuNPs using *E. milii* des moul extract is a novel approach and the observed characteristics, including the distinct surface-plasmon-resonance band, surface potential, and size distribution, align with established literature on biogenic nanoparticle synthesis. The G-CuNPs demonstrated fungicidal potency at 5 mg/ml concentration against clinically isolated *C. albicans*. Previous reports also validate the effective antifungal efficacy of green synthesized copper nanoparticles against *Candida* species [ 27 ]. G-CuNPs exhibit impressive antifungal potential through various mechanisms, such as the generation of free radical species, which could damage the cells and induce apoptosis, peroxidation of lipids, or removal of antioxidant enzymes, like glutathione [ 28 ]. The G-CuNPs took only 3 h to completely eliminate the *C. albicans*, and a high level of intracellular ROS generation and DNA degradation was observed in tested strains supplemented with G-CuNPs. The G-CuNPs may interact with the sulfur-containing proteins in the cell wall, leading to irreversible changes that ultimately cause cell death [ 29 ]. The G-CuNPs disrupted the cell wall which is also another reason for bacterial cell death [ 30 ]. Another study revealed that increased uptake of G-CuNPs has negative surface charges [ 13 ]. The NPs with a high negative surface charge strongly react with microbial cells or mammalian cells, causing fluidity in the cell membrane and leading to the gelation of membranes [ 32 ]. The electrostatic interaction among negatively charged NPs with the phosphate group increases surface tension resulting in pores formation in the cell wall [ 33 ]. Apart from this, other factors also contribute to its effectiveness, such as the shape and size of the synthesized nanoparticle, type of reducing agents used in the synthesis, and the approach of the synthesis [ 34 ]. The suggestion of using G-CuNPs in antimicrobial coatings for indwelling medical devices logically follows the observed antifungal activity, addressing the therapeutic complication posed by the increased prevalence of candidemia among ICU patients [ 13 ].

## Conclusion

An intensive care setting is a high-risk area for acquiring infection. The high rate of candidemia in ICUs is due to frequent use of medical devices, increased device days, length of hospital stays, patient disease severity, and immunocompromised state. This study highlighted the need for a context-specific approach to infection control and management. Synthesis of G-CuNPs using *E. milii* des moul extract presents a promising approach. The fungicidal potency of G-CuNPs against clinically isolated *C. albicans* suggests a potential breakthrough in combating microbial colonization. Current results suggest incorporating G-CuNPs into antimicrobial coatings for medical devices to prevent infections associated with medical interventions. The findings serve as a catalyst for further research and clinical exploration.

## References

[1] Bajpai VK, Khan I, Shukla S, Kumar P, Rather IA, Park YH, Huh YS, Han YK ( 2019). Invasive fungal infections and their epidemiology: Measures in the clinical scenario. Biotechnology and Bioprocess Engineering.

[2] Negm EM, Mohamed MS, Rabie RA, Fouad WS, Beniamen A, Mosallem A, Tawfik AE, Salama HM ( 2023). Fungal infection profile in critically ill COVID-19 patients: a prospective study at a large teaching hospital in a middle-income country. BMC Infect Dis.

[3] Haque M, Sartelli M, McKimm J, Bakar MA ( 2018). Health care-associated infections–an overview. Infect Drug Resist.

[4] Kotey FC, Dayie NT, Tetteh-Uarcoo PB, Donkor ES ( 2021). Candida bloodstream infections: changes in epidemiology and increase in drug resistance. Infect Dis (Auckl)..

[5] Ahmad S, Kumar S, Rajpal K, Sinha R, Kumar R, Muni S, Kumari N ( 2022). Candidemia Among ICU Patients: Species Characterisation, Resistance Pattern and Association With Candida Score: A Prospective Study. Cureus.

[6] Barantsevich N, Barantsevich E ( 2022). Diagnosis and treatment of invasive candidiasis. Antibiotics.

[7] Fang W, Wu J, Cheng M, Zhu X, Du M, Chen C, Liao W, Zhi K, Pan W ( 2023). Diagnosis of invasive fungal infections: challenges and recent developments. J Biomed Sci.

[8] Salam MA, Al-Amin MY, Salam MT, Pawar JS, Akhter N, Rabaan AA, Alqumber MA ( 2023). Antimicrobial resistance: a growing serious threat for global public health. In Healthcare.

[9] Amjad R, Mubeen B, Ali SS, Imam SS, Alshehri S, Ghoneim MM, et al ( 2021). Green synthesis and characterization of copper nanoparticles using Fortunella margarita leaves. Polymers.

[10] Ortiz B, Aguilar K, Galindo C, Molina L, Fontecha G ( 2022). Candida species isolated from clinical samples in a tertiary hospital in Honduras: Where is Candida auris?. Curr Med Mycol.

[11] Sheppard DC, Locas MC, Restieri C, Laverdiere M ( 2008). Utility of the germ tube test for direct identification of Candida albicans from positive blood culture bottles. J Clin Microbiol.

[12] Melhem MS, Bertoletti A, Lucca HR, Silva RB, Meneghin FA, Szeszs MW ( 2013). Use of the VITEK 2 system to identify and test the antifungal susceptibility of clinically relevant yeast species. Braz J Microbiol.

[13] Kaur N, Shriwastav S, Dev J, Aman S, Hassan M, Kumar A, Bala R, Singh M ( 2023). Mechanistic insights of Euphorbia milii des moul mediated biocompatible and non-cytotoxic, antimicrobial nanoparticles: an answer to multidrug resistant bacteria. World J Microbiol Biotechnol.

[14] Yang HC, Mikami Y, Yazawa K, Taguchi H, Nishimura K, Miyaji M, Branchini ML, Aoki FH, Yamamoto K ( 1998). Colorimetric MTT assessment of antifungal activity of D0870 against fluconazole-resistant Candida albicans. Mycoses.

[15] Aman S, Kaur N, Mittal D, Sharma D, Shukla K, Singh B, Sharma A, Siwal SS, Thakur VK, Joshi H, Gupta R ( 2023). Novel biocompatible green silver nanoparticles efficiently eliminates multidrug resistant nosocomial pathogens and mycobacterium species. Indian J Microbiol.

[16] Aman S, Mittal D, Shriwastav S, Tuli HS, Chauhan S, Singh P, et al ( 2022). Prevalence of multidrug-resistant strains in device associated nosocomial infection and their in vitro killing by nanocomposites. Ann Med Surg (Lond)..

[17] Barbara D. Alexander, Gary W. Procop, Philippe Dufresne, Jeff Fuller, Mahmoud A. Ghannoum, Kimberly E. Hanson, et al ( 2017). Reference Method for Broth Dilution Antifungal Susceptibility Testing of Yeasts, 4th Edition.

[18] Kaur H, Singh S, Rudramurthy SM, Ghosh AK, Jayashree M, Narayana Y, Ray P, Chakrabarti A ( 2020). Candidaemia in a tertiary care centre of developing country: Monitoring possible change in spectrum of agents and antifungal susceptibility. Indian J Med Microbiol.

[19] Tak V, Mathur P, Varghese P, Gunjiyal J, Xess I, Misra MC ( 2014). The epidemiological profile of candidemia at an Indian trauma care center. J Lab Physicians.

[20] Gandham NR, Vyawahare CR, Jadhav SV, Misra RN ( 2016). Candidemia: Speciation and antifungal susceptibility testing from a tertiary care hospital in Maharashtra, India. Medical Journal of Dr. DY Patil University.

[21] Gautam G, Rawat D, Kaur R, Nathani M ( 2022). Candidemia: Changing dynamics from a tertiary care hospital in North India. Curr Med Mycol.

[22] Ahmed S, Shahid M, Fatima N, Khan F, Tayyaba U ( 2020). Candidemia–Changing trends from Candida albicans to non-albicans Candida from a tertiary care center in western UP, India. CHRISMED Journal of Health and Research.

[23] Rajni E, Chaudhary P, Garg VK, Sharma R, Malik M ( 2022). A complete clinico-epidemiological and microbiological profile of candidemia cases in a tertiary-care hospital in Western India. Antimicrob Steward Healthc Epidemiol.

[24] Popović S, Begović-Kuprešanin V ( 2023). Factors’ analysis associated with adverse outcome of the treatment of patients with invasive candidiasis. Srpski arhiv za celokupno lekarstvo.

[25] Baghdadi E, Khodavaisy S, Rezaie S, Abolghasem S, Kiasat N, Salehi Z, Sharifynia S, Aala F ( 2016). Antifungal Susceptibility Patterns of Candida Species Recovered from Endotracheal Tube in an Intensive Care Unit. Adv Med.

[26] Ying S, Guan Z, Ofoegbu PC, Clubb P, Rico C, He F, Hong J ( 2022). Green synthesis of nanoparticles: Current developments and limitations. Environmental Technology & Innovation.

[27] Muñoz-Escobar A, Reyes-López SY ( 2020). Antifungal susceptibility of Candida species to copper oxide nanoparticles on polycaprolactone fibers (PCL-CuONPs). PLoS One.

[28] Naz S, Gul A, Zia M ( 2020). Toxicity of copper oxide nanoparticles: a review study. IET nanobiotechnology.

[29] Kaur R, Kaur K, Alyami MH, Lang DK, Saini B, Bayan MF, Chandrasekaran B ( 2023). Combating microbial infections using metal-based nanoparticles as potential therapeutic alternatives. Antibiotics.

[30] Ma X, Zhou S, Xu X, Du Q ( 2022). Copper-containing nanoparticles: Mechanism of antimicrobial effect and application in dentistry-a narrative review. Front Surg.

[31] Chung K, Bang J, Thacharon A, Song HY, Kang SH, Jang WS, et al ( 2022). Non-oxidized bare copper nanoparticles with surface excess electrons in air. Nat Nanotechnol.

[32] Foroozandeh P, Aziz AA ( 2018). Insight into cellular uptake and intracellular trafficking of nanoparticles. Nanoscale Res Lett.

[33] Behzadi S, Serpooshan V, Tao W, Hamaly MA, Alkawareek MY, Dreaden EC, Brown D, Alkilany AM, Farokhzad OC, Mahmoudi M ( 2017). Cellular uptake of nanoparticles: journey inside the cell. Chem Soc Rev.

[34] Chakraborty N, Banerjee J, Chakraborty P, Banerjee A, Chanda S, Ray K, Acharya K, Sarkar J ( 2022). Green synthesis of copper/copper oxide nanoparticles and their applications: a review. Green Chemistry Letters and Reviews.

